# Risk Factors in Cardiovascular Disease in Systemic Lupus Erythematosus

**DOI:** 10.2174/157340313805076304

**Published:** 2013-02

**Authors:** Nailú Angélica Sinicato, Priscila Aparecida da Silva Cardoso, Simone Appenzeller

**Affiliations:** Department of Medicine, Rheumatology Unit, Faculty of Medical Science, State University of Campinas, Brazil

**Keywords:** Nontraditional risk factors, cardiovascular disease, systemic lupus erythematosus.

## Abstract

Systemic lupus erythematosus (SLE) is a chronic and multisystemic autoimmune disorder which predominantly affecting women. The most common cause of death in SLE patients affected by disease for more than 5 years is cardiovascular disease (CVD). Epidemiological observations suggest that, together with classical conventional risk factors, other mechanisms (non-conventional/disease-specific factors) promote accelerated atherosclerosis in inflammatory diseases like SLE. Traditional CVD risk factors included age, hypertension, diabetes mellitus, dyslipidemia, previous vascular event defined as previous history of cerebrovascular accidents or ischemic heart disease, menopause and smoking. The non-traditional factors presents in SLE are disease-specific like renal disease manifestation as Lupus nephritis (LN), presence of pro-inflammatory cytokines, some of inflammatory mediators, antiphospholipid antibodies, anti-oxLDL antibodies, corticosteroid uses and cumulative dose of glucocorticoids. We will review traditional and non-traditional risk factors associated with CVD in SLE patients.

## INTRODUCTION

Systemic lupus erythematosus (SLE) is a chronic and multisystemic disorder linked to loss of immune tolerance to self-antigens and the production of a variety of autoantibodies predominantly affecting women of childbearing age [1, 2]. 10–20% of all SLE cases occur approximately in the first two decades of life [3]. It’s course is characterized by periods of exacerbation and remission with breakouts difficultly to be controlled. The most common cause of death in SLE patients affected by disease for more than 5 years is cardiovascular disease (CVD) [4].

Coronary artery disease (CAD) is one of the cardiovascular manifestations observed in young SLE patients. The clinical manifestations of CAD in SLE can result from several pathophysiologic mechanisms, including atherosclerosis, arteritis, thrombosis, embolization, spasm, and abnormal coronary flow [5,6].

The striking clinical characteristic of most patients with SLE who have a myocardial infarction is their young age. This demographic characteristic suggests that patients with SLE are at increased risk of myocardial infarction and that reports of myocardial infarction in patients with SLE do not simply represent chance occurrences. Fatal myocardial infarction has been reported to be 3 times higher in patients with SLE than in age- and gender -matched control subjects [6-8]. Recent case- control series have confirmed that the risk of myocardial infarction in patients with SLE is increased between 9- and 50-fold over that in the general population [6, 7, 9]. It has been increasingly recognized that patients with SLE have a high cardiovascular mortality.

The impact of coronary heart disease (CHD) on morbidity and mortality in patients with established SLE has assumed increasing importance in their long-term management. SLE is a chronic inflammation of organism and inflammation is a prominent feature of atherosclerotic lesions [4]. To prove CVD features in SLE we observed the prevalence of clinically manifest ischemic heart disease has ranged between 8% and 16% in various studies [10-13].

Clinical epidemiological observations strongly suggest that, together with classical conventional risk factors, other mechanisms (non-conventional/disease-specific factors) promote accelerated atherosclerosis in inflammatory diseases like SLE [8,9,14-16]. SLE is now considered to be an independent risk factor for the development of atherosclerosis. Viewing atherosclerosis as an inflammatory disease, this association becomes stronger and better understood.

## TRADITIONAL RISK FACTORS

**Fig. (1):**
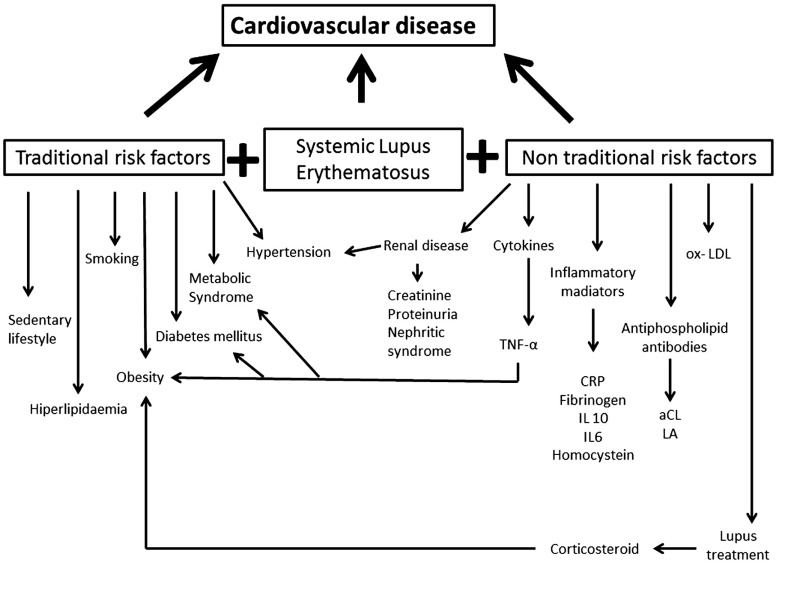
Traditional and non-traditional risk factors for cardiovascular disease in SLE.

Traditional CVD risk factors included age, hypertension, diabetes mellitus, dyslipidemia, previous vascular event defined as previous history of cerebrovascular accidents or ischemic heart disease, menopause and smoking [17]. Among these factors hypertension, dyslipidemia and hypercholesterolemia have been shown to be more prevalent in SLE [18] (Fig. **1**). Metabolic syndrome (MetS) is considered an independent predictor of cardiovascular morbidity and mortality that identifies substantial additional cardiovascular risk beyond the sum of the individual risk factors. In addition to the cardiovascular risk factors that comprise the metabolic syndrome, there is a strong relationship with inflammation [19, 20]. Several studies have shown that the prevalence of MetS is increased in SLE [21-25].

On important finding is that SLE patients have an increased risk for cardiovascular events even after adjustment for Framingham risk factors (hypertension, hypercholesterolemia, diabetes mellitus, older age, and postmenopausal status) [7], so it is necessary to develop other methods to determine the subgroup of SLE patients that are at highest risk for CVD disease. However, traditional CV risk factors alone cannot explain the excess risk of premature CV disease among lupus patients and this suggests that disease-related factors constitute an equal or even greater risk (Fig. **1**).

## NON-TRADITIONAL RISK FACTORS

### Renal Manifestations

The non-traditional factors presents in SLE are Lupus-specific (Fig. **1**). Renal disease manifestation like Lupus nephritis (LN) is known to be one of the important factors for accelerated atherosclerosis in SLE [26–29]. Studies had showed that increasing level of serum creatinine and the presence of proteinuria were strongly associated with patients with CVD [30-33]. Elevated serum creatinine and proteinuria indicate renal impairment to a certain extent, which may present as nephritic syndrome. It was reported that nephritic syndrome and excess proteinuria were related to prothrombotic risk, which might lead to the development of clinical CVD [33,34].

### Cytokines

Pro-inflammatory cytokines released as a result of the chronic systemic inflammation associated with SLE are involved in CVD. Tumoral necrosis factor alpha (TNF-α) which may act in an autocrine manner to modification insulin transduction inhibiting glucose transport, causing in elevated levels, insulin resistance [35]. Studies about TNF-α administration showed that this treatment can causes an increase serum level of triglycerides and very low density lipoproteins in rats and humans [36-38].

SLE patients presents high TNF-α levels, one of the main inhibitors of adipocytokines production; however it was noted that there is an increase in adipocytokine mainly in SLE patients with renal involvement regardless of the TNF- α of the patient [39].

It is known that inflammatory cytokines can stimulate the hypothalamic-pituitary-adrenal (HPA), resulting in an increase in glucocorticoid levels that will affect some immune and inflammatory processes [40,41].

### Inflammatory Mediators

Some of inflammatory mediators are associated to atherosclerosis, such as: overproduction of c-reactive protein (CRP) a protein that appears in systemic inflammation and can be a strong predictor for CVD [42], fibrinogen, and interleukins; IL-10 which has an atheroprotective function, IL-6 one of the most potent proinflammatory cytokines which stimulate the release of fatty acids, and it’s associated with increased cardiovascular mortality and prognosis in the general population [43]. 

Homocystein is a prothrombotic coagulation factor, that has a toxic effect on endothelium, increases collagen production and decrease nitric oxide availability [9]. Homocysteinemia is considered a new risk factor on atherosclerosis development in SLE patients [9].

## ANTIPHOSPHOLIPID ANTIBODIES

Antiphospholipid antibodies are a heterogeneous group of autoantibodies, including, anticardiolipin antibody (aCL) and lupus anticoagulant (LA), generally directed to phospholipid binding proteins; in this regard, β2GPI represents the major antigenic target [44]. LA has been associated with angina and myocardial infarction [6-47], as well as anti - oxLDL antibodies elevated levels [4,6,31,48,49].

## ANTI-OXIDIZED LOW-DENSITY LIPOPROTEIN (OXLDL)

During early atherogenesis, LDL become trapped in the subendothelial space and is subsequently oxidized [50,51]. This oxLDL increases the adhesive properties of endothelial cells and induces the activation of monocytes and T cells and is thought to be responsible for triggering inflammatory responses in macrophages and vascular wall cells [52-54]. This oxLDL is able to take up macrophages and other cells in the atherosclerotic plaque and develop them into foam cells [52]. Anti-oxLDL antibodies are present in patients with atherosclerosis, independently of its etiology [55].

## LUPUS TREATMENT

As the antiinflammatory/immunosuppressive treatment of patients with SLE continues to improve [56], the contribution of CVD to morbidity and mortality is likely to increase [57]. 

Corticosteroid uses has favorable effect on reducing disease activity and inflammation, but the cumulative dose of glucocorticoids promote hypertriglyceridemia and insulin resistance and are associated with a higher cholesterol plasma level, higher blood pressure and weight change in lupus patients [58].

Hydroxychloroquine has several protective effect, including effects on the reducing serum lipid profile, increase HDL, reduces the insulin resistance and inhibition of platelet aggregation in SLE [57].

Studies suggests that patients that received early treatment of the disease with pulse IV methylprednisolone to achieve remission, had a lower systolic and diastolic blood pressure, total cholesterol and triglyceride levels proving how it is important in reducing the CV risk among these patients [11,17].

It’s too early to say that mycophenolic acid had an antiatherogenic effect, but recently, studies had examined its potential in view of its multiple roles in inhibiting multiple inflammatory mediators and lymphocytes, particularly T cells and macrophages which play major roles in atherogenesis [17,59].

## CONCLUSION

In conclusion, in addition to traditional risk factors SLE patients have several disease related risk factors that explain increase CVD. A careful control for this risk factors is essential to continuously improve survival in SLE.

## GRANTS

Fundação Amparo À Pesquisa Estado São Paulo-Brasil (FAPESP 2008/02917-0 and 2009/06049-6 and 2009/11076-2), Conselho Nacional Pesquisa Desenvolvimento-Brasil CNPq (300447/2009-4)

## DISCLOSURES

The authors don’t have any conflict of interest; This is an update froma previous version published in this journal Cardiovascular disease in systemic lupus erythematosus: The role of traditional and lupus related risk factors. CCR 2008; 4: 2: 116-122.
